# A case report of atrial fibrillation with abdominal aortic embolism mimicking acute myelitis

**DOI:** 10.1097/MD.0000000000048398

**Published:** 2026-04-17

**Authors:** Tong Liu, Yuanyuan Che

**Affiliations:** a Clinical Medical College (Affiliated Hospital), Jining Medical University, Jining, Shandong, P.R. China; b Department of Cardiology, Zaozhuang Municipal Hospital, Zaozhuang, Shandong, P.R. China.

**Keywords:** abdominal aortic embolism, atrial fibrillation, myelitis

## Abstract

**Rationale:**

The most common complication of atrial fibrillation is cerebral embolism, while abdominal aortic embolism is relatively rare. This case highlights the need for heightened clinical awareness of systemic embolic complications associated with atrial fibrillation.

**Patient concerns:**

A 76-year-old male patient presented with sudden onset of bilateral lower limb weakness lasting 12 hours, accompanied by numbness and pain in both legs. Symptoms have persisted without improvement and are associated with gross hematuria.

**Diagnoses:**

Computed tomography angiography of the chest and abdomen reveals occlusion of the lower abdominal aorta (below the renal arteries) and bilateral lower extremity arteries; thrombus formation in the descending aorta; incidental findings of low-density areas in both renal parenchyma.

**Interventions:**

Administer enoxaparin for anticoagulation. Recommend transferring the patient to an intensive care unit or a higher-level hospital for treatment. The patient and their family are aware of the associated risks and have declined further treatment.

**Outcomes:**

During a subsequent follow-up phone call, it was reported that the patient had passed away.

**Lessons:**

This case demonstrates that complications of atrial fibrillation are not limited to common cerebral embolism; embolism involving other organs can also be fatal.

## 1. Introduction

The most common complication of atrial fibrillation is cerebral embolism. This paper reports a relatively rare case of atrial fibrillation complicated by abdominal aortic embolism. We review the diagnostic and therapeutic process and relevant literature to promote early diagnosis and treatment of this condition in clinical practice. This aims to enhance understanding of atrial fibrillation, facilitate systematic diagnosis and treatment, and improve patient outcomes.

## 2. Case report

A 76-year-old male patient presented with “sudden bilateral lower limb weakness for over 12 hours.” He has a 10-year history of “heart disease” treated with “Suoxiaojiuxin Pills” (a compound traditional Chinese medicine formulation primarily composed of Chuanxiong, mainly used to relieve angina pectoris) denying other medical conditions. He has a 50-year history of smoking and alcohol consumption. Approximately 12 hours prior, the patient experienced sudden onset of bilateral lower limb weakness without apparent precipitating factors, accompanied by numbness and pain in both legs. He was evaluated at a local health center without a definitive diagnosis. Symptoms persisted without improvement, leading to presentation at our hospital’s emergency stroke clinic. Subsequent computer tomography scans of the thoracic and lumbar spine, along with magnetic resonance imaging examinations of the brain, thoracic spine, cervical spine, and lumbar spine, primarily indicated lumbar spinal stenosis. A consultation with the Spine Surgery Department was requested. After reviewing the imaging studies, no significant signs of spinal cord compression were observed at the corresponding segments. The possibility of spinal cord compression causing weakness in both lower limbs was ruled out, and the patient was admitted to Neurology.

*Physical examination*: Alert and oriented, blood pressure 131/92 mm Hg. Clear breath sounds bilaterally without rales. Heart rate 113 bpm, absolute arrhythmia. Strong heart sounds; no murmurs heard in all valve auscultation areas. Abdominal wall soft, no tenderness or rebound tenderness. Skin mottling scattered over both lower limbs, low skin temperature. Dorsal foot pulses not palpable in both lower limbs. No edema or varicose veins in both lower limbs. Muscle strength grade 0 in both lower limbs. Abdominal wall reflex, knee reflex, and Achilles tendon reflex all elicited. Bilateral Babinski sign negative. Increased muscle tone in right lower limb, normal in left lower limb. Deep and superficial sensation absent in both lower limbs; sensory level below T11. No bowel movement; indwelling catheter showed gross hematuria.

**Figure 1.**
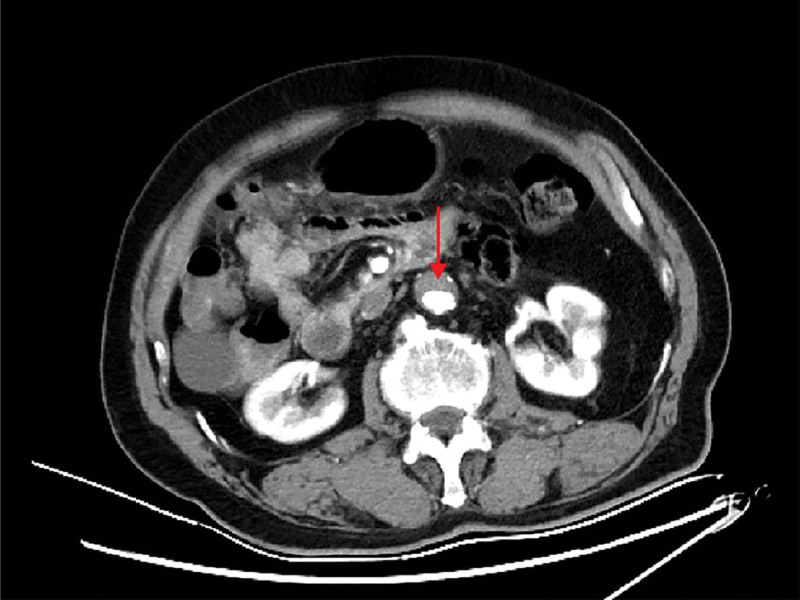
Computed tomography angiography of the chest and abdomen revealed atherosclerosis of the abdominal aorta with thrombus formation.

**Figure 2.**
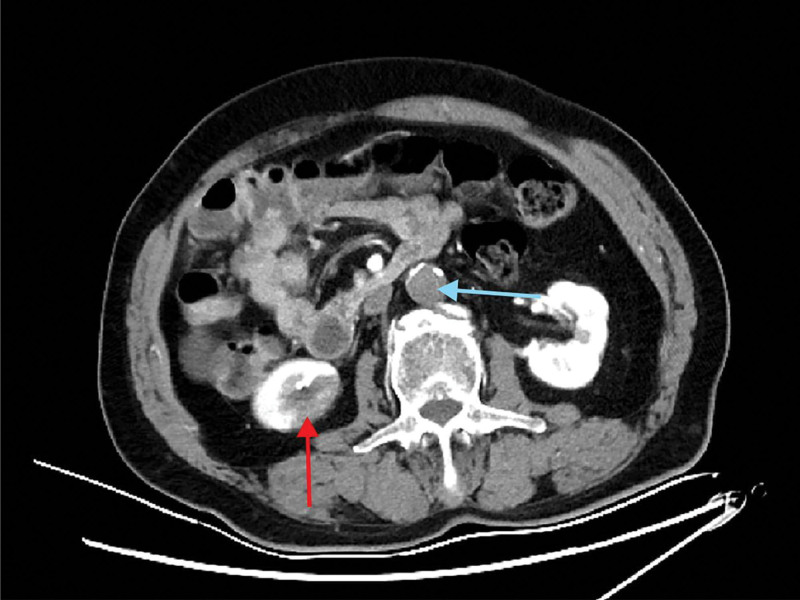
Computed tomography angiography of the chest and abdomen reveals thrombus formation and occlusion within the abdominal aorta (blue arrow); localized infarction of the lower pole of the right kidney (red arrow).

**Figure 3.**
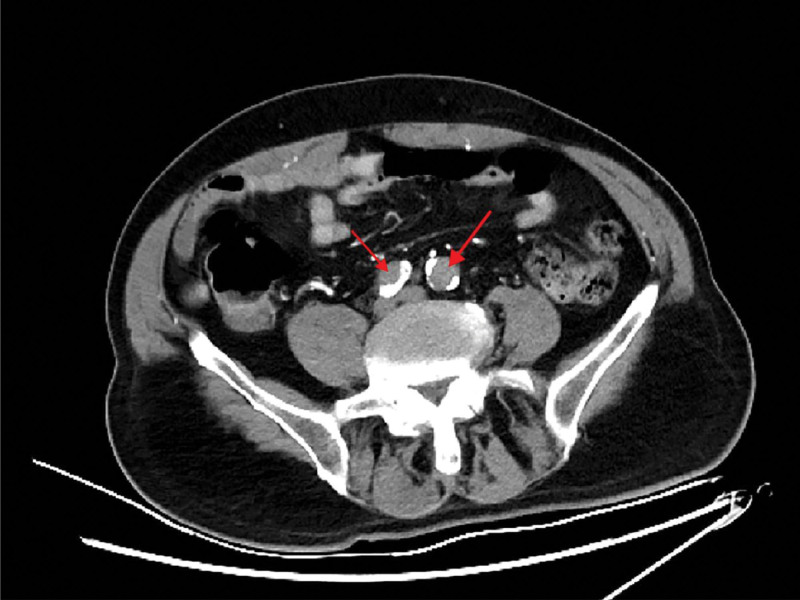
Computed tomography angiography of the chest and abdomen revealed bilateral occlusion of the common iliac arteries.

**Figure 4.**
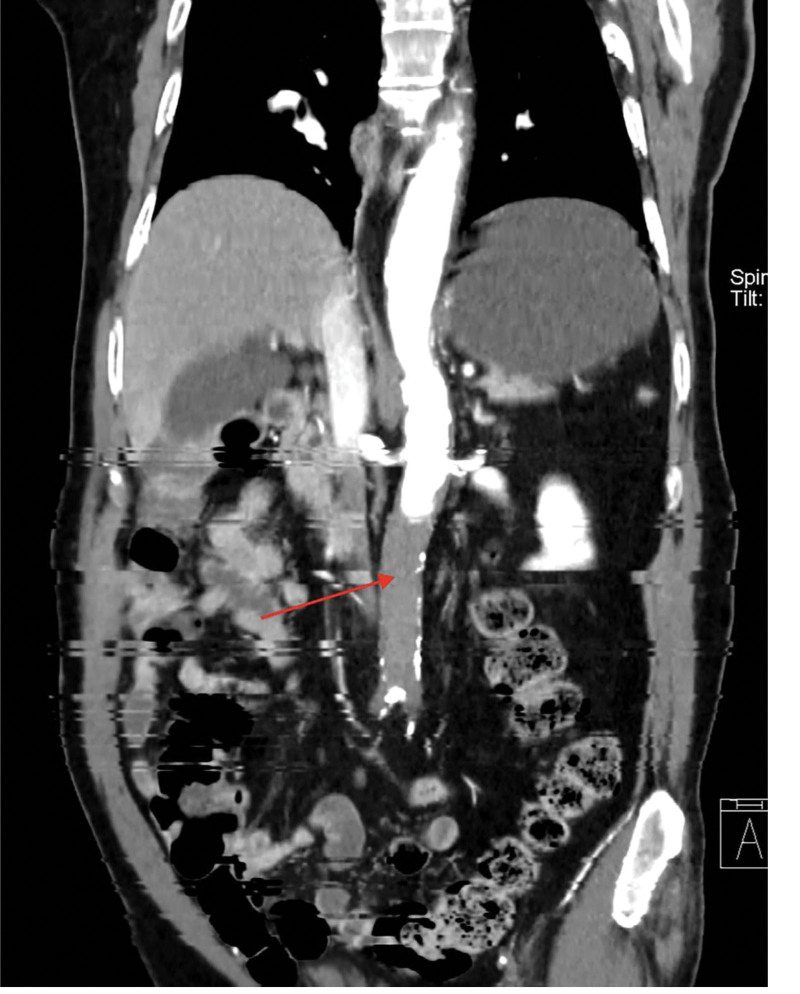
Computed tomography angiography of the chest and abdomen (coronal view) demonstrates occlusion of the abdominal aorta (below the level of the renal artery origins).

**Figure 5.**
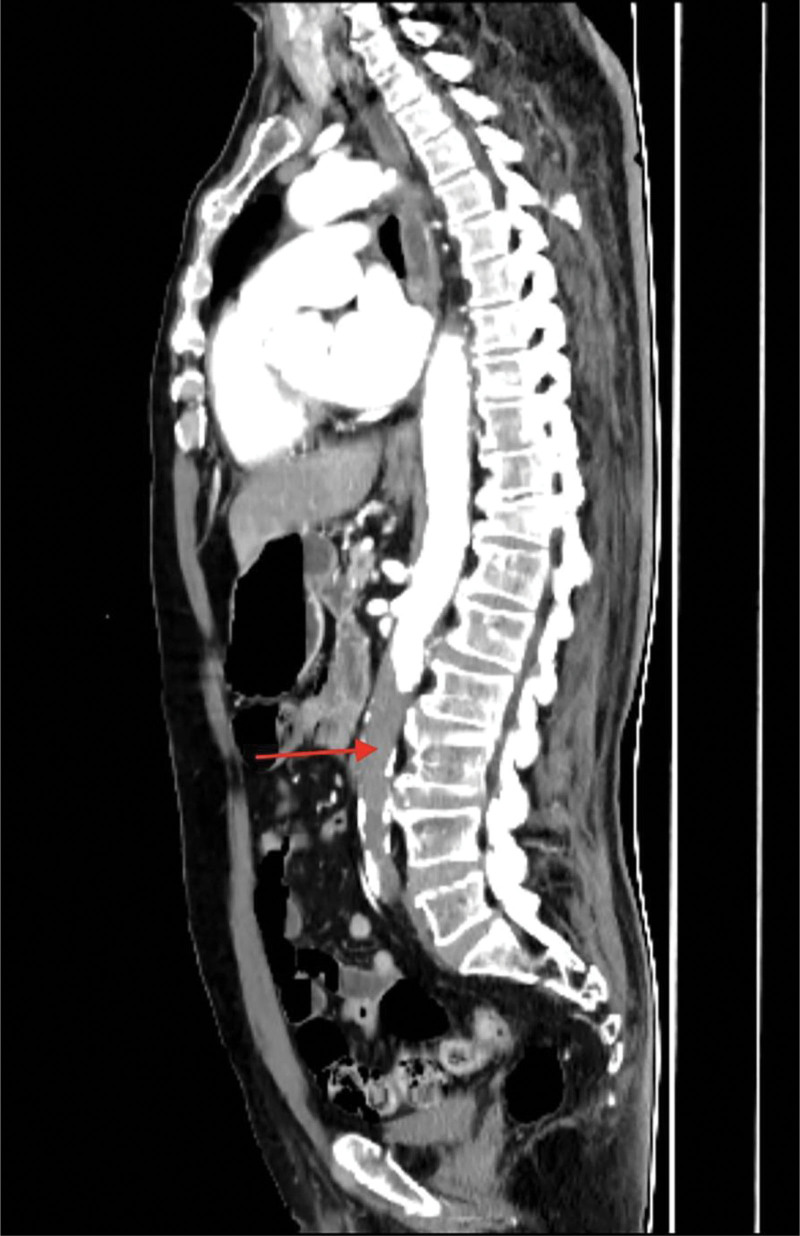
Computed tomography angiography of the chest and abdomen (sagittal view) demonstrates occlusion of the descending aorta.

**Figure 6.**
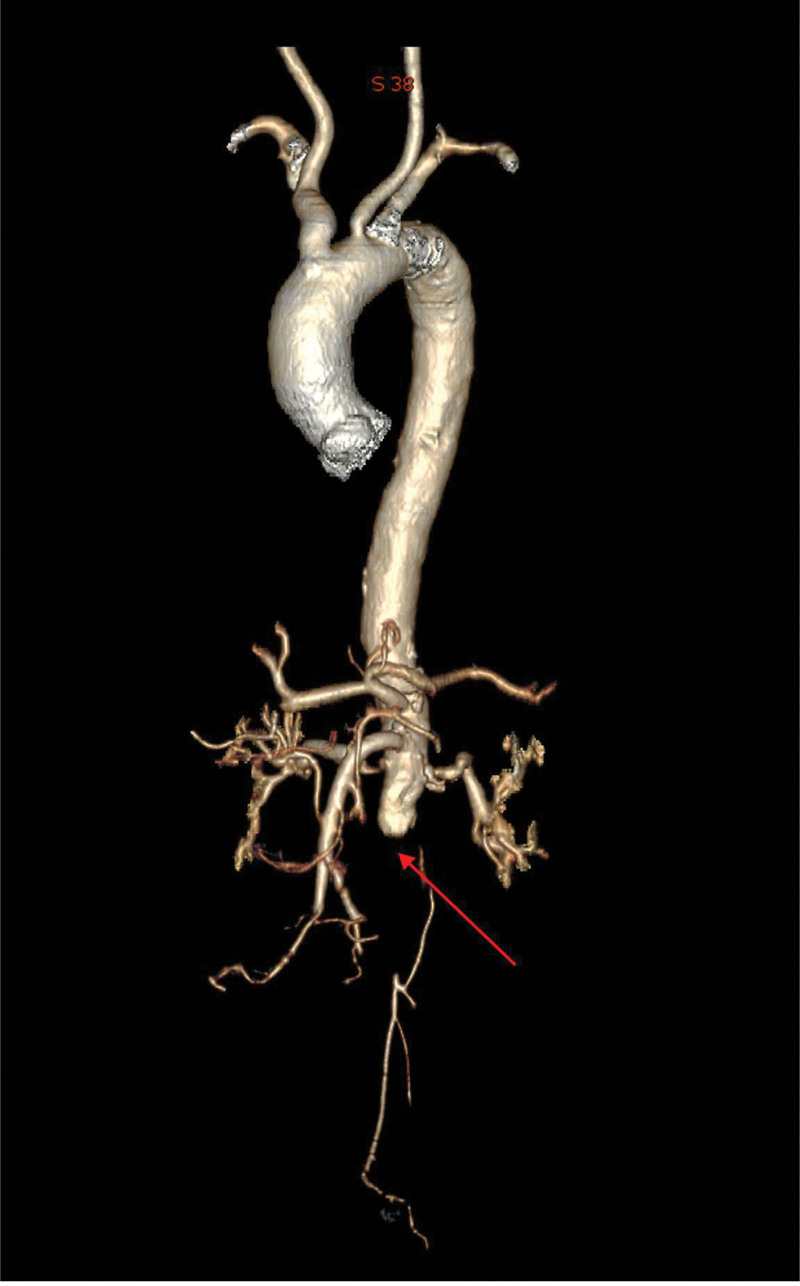
Three-dimensional imaging demonstrates no visualization of the descending abdominal aorta (below the renal arteries) and bilateral iliac arteries.

**Figure 7.**
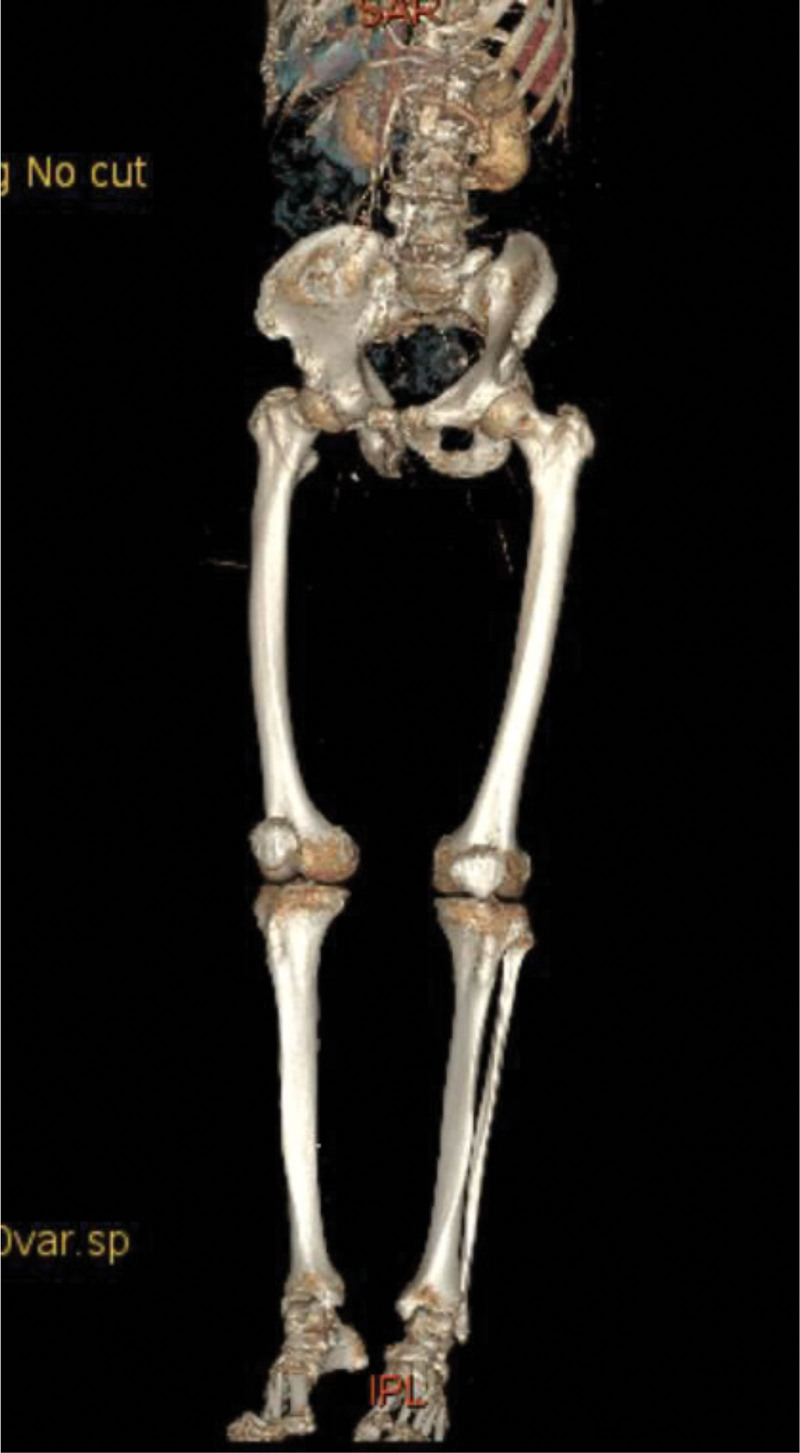
Three-dimensional imaging demonstrates no visualization of the bilateral lower limb arteries.

Abnormal laboratory findings included: myoglobin: 964.7 ng/mL, glucose: 12.88 mmol/L, D-dimer: 5270 ng/mL, white blood cells: 12.88 × 10^9^/L, neutrophil percentage: 91.8%. Upon admission, the primary diagnosis was limb weakness, pending further investigation. Due to diagnostic uncertainty, the attending physician conducted a detailed physical examination and ordered a thoracoabdominal computed tomography angiography (CTA). The primary findings revealed: occlusion of the inferior abdominal aorta (below the renal arteries) and bilateral lower limb arteries; thrombus formation in the descending aorta; bilateral areas of low density in the renal parenchyma (Figs. 1–7).

A vascular surgery consultation was requested, recommending anticoagulation therapy after excluding contraindications. The patient’s family was informed of the condition and the bleeding risks associated with anticoagulation. Following consent, enoxaparin was administered for anticoagulation. The patient was advised to transfer to an intensive care unit or a higher-level hospital for treatment. The patient and family acknowledged the associated risks, declined further treatment, and requested discharge.

## 3. Discussion and conclusion

### 3.1. Differential diagnosis

The patient presented with “bilateral lower limb weakness.” In summary, the diagnostic process is as follows: onset of symptoms → initial imaging → neurological admission → CTA confirmation. Based on the preliminary diagnosis: emergency laboratory tests did not reveal hypokalemia, ruling out weakness caused by hypokalemia. Although lumbar spine pathology was present, consultation with a spine surgeon indicated no significant signs of spinal cord compression at the corresponding segment. Acute cerebrovascular disease: while the patient presented with bilateral lower limb weakness, cerebrovascular events typically cause unilateral hemiplegia. Bilateral lower limb paralysis occurs only in exceptional cases, such as brainstem involvement or extensive bilateral infarction/hemorrhage.

The patient’s emergency cranial magnetic resonance imaging showed no acute ischemic or hemorrhagic lesions. Due to the predominance of neurological symptoms, acute limb ischemia is often misdiagnosed in its early stages, necessitating differential diagnosis primarily from neurological disorders. The patient’s symptoms closely resemble acute myelitis. Acute myelitis typically progresses to its peak within hours or days, initially presenting as bilateral lower limb weakness and gait disturbance, rapidly advancing to complete paraplegia with a sensory level.^[1]^ This patient’s presentation aligns well with this pattern. This condition must also be differentiated from Guillain-Barré syndrome. Its primary clinical features include acute onset, with most patients reporting a history of preceding infection. The classic form typically presents with quadriplegia, while the paraplegic variant manifests as lower limb weakness with diminished or absent tendon reflexes. Exclusion criteria include a clearly defined sensory level.^[2]^ This patient reported no relevant infection history and meets exclusion criteria, allowing temporary exclusion of this diagnosis; myasthenia gravis is an autoimmune disorder typically presenting with fluctuating weakness and fatigue that worsens with activity and improves with rest.^[3]^ The patient had an acute onset without marked symptom fluctuation, instead exhibiting persistent, unrelieved weakness. During diagnosis and treatment, differential diagnosis with acute myelitis was prioritized. Due to the patient’s brief overall illness duration and time constraints, cerebrospinal fluid examination was not performed. However, beyond myelitis symptoms, the patient exhibited gross hematuria, decreased skin temperature, diminished dorsalis pedis pulses, mottled skin, and pain (manifestations not explainable by myelitis alone).

### 3.2. Analysis of clinical presentation

Based on symptoms and signs, the patient exhibited Grade 0 muscle strength in the lower limbs accompanied by pain, numbness, and mottled skin. Clinicians suspected compromised limb perfusion with potential ischemic necrosis. Indwelling catheterization revealed gross hematuria, raising suspicion of renal infarction and myoglobinuria due to rhabdomyolysis. On admission, physical examination revealed atrial fibrillation rhythm. Given that atrial fibrillation can cause hemodynamic instability, combined with elevated D-dimer and myoglobin levels in laboratory tests, a strong suspicion of thrombosis arose. Thoracic and abdominal CTA was performed, confirming the diagnosis. While cerebral infarction is a common complication of atrial fibrillation, concomitant embolism involving the abdominal aorta and multiple organ vessels is rare. Clinically, this patient presented a challenging diagnosis prone to misdiagnosis and inappropriate treatment. Reviewing the findings against the patient’s presentation, the primary clinical manifestations of acute limb ischemia are the “6P signs”: pain, pallor, pulselessness, paresthesia, paralysis, and skin temperature changes.^[4]^ These align with the patient’s clinical presentation. However, as an internist, one might not promptly recognize these as signs of limb ischemia.

### 3.3. Clinical reflection

The patient reported a history of “heart disease” and had been taking “Suoxiaojiuxin Pills” for treatment. The admission physical examination revealed atrial fibrillation, though the patient was unaware of this condition and had never undergone systematic diagnosis or treatment. Based on clinical diagnostic clues for atrial fibrillation obtained from the physical examination, the physician determined that thrombus formation was highly likely related to cardiogenic embolism. Although the family declined further testing, the patient’s prolonged illness duration and lack of anticoagulant therapy provided a reasonable clinical explanation for the extensive thrombus formation.The patient’s long-term smoking history likely contributed to poor vascular condition. Additionally, significant delay in treatment led to extensive thrombus formation. Overall, the patient presented with severe disease and prolonged delay (over 12 hours had elapsed since symptom onset at admission). Throughout the course of illness, gradual occlusion of the abdominal aorta progressively obstructed blood flow, causing ischemia in related organs. This resulted in ischemia of the spinal cord, kidneys, and muscles in both lower limbs. This subsequently led to rhabdomyolysis and even necrosis, causing massive myoglobin and its degradation products to obstruct and damage renal tubules. This ultimately resulted in acute renal failure, posing a life-threatening condition.^[5]^

Once abdominal aortic embolism occurs, the primary treatment is prompt surgical thrombectomy to restore perfusion within 12 hours, combined with low molecular weight heparin anticoagulation therapy.^[5]^ Although this patient underwent thoracoabdominal CTA within the shortest possible time after admission for definitive diagnosis, the condition was extremely critical with severely limited treatment options, resulting in anticoagulation therapy alone. However, even when anticoagulation is initiated immediately after detecting atrial fibrillation and the optimal international normalized ratio is achieved, thrombus formation may still occur during anticoagulation therapy.^[6]^ A 2023 case report described a patient with a straddling abdominal aortic embolism complicated by multiple visceral artery embolisms.^[7]^ Despite aggressive interventions including abdominal aorta resection, bilateral iliac artery thrombus removal, and thrombolysis, renal function failed to recover. Persistent hemodialysis could not clear excessive myoglobin and inflammatory mediators, and blood pressure remained uncontrollable throughout the course, leading to discontinuation of treatment.^[7]^ Another patient admitted with “abdominal pain and bilateral lower limb weakness for over 2 hours” underwent emergency abdominal aortography and catheter placement. Despite urgent thrombolysis with urokinase, low molecular weight heparin, and warfarin, bilateral lower limb skin temperature and mottling showed no improvement after approximately 3 hours. Following consultation, 3 thrombectomy procedures were performed. Postoperatively, the patient received adjunctive anti-infective therapy, anticoagulation, adequate hydration, urine alkalinization, and diuresis, leading to favorable recovery.^[8]^

The diagnostic and therapeutic process in this case was limited, primarily due to delayed presentation. The patient had been experiencing symptoms for over 12 hours at admission. Although the diagnosis was confirmed shortly after admission, the golden window for reperfusion therapy had already been missed. While physical examination suggested atrial fibrillation, key etiological investigations (such as electrocardiogram and echocardiography) could not be performed due to family refusal. Review of the literature indicates that ultra-early intervention (surgical/thrombolytic) is crucial for improving prognosis. However, in this case, the prolonged disease course led to extensive thrombus formation, resulting in high risks and uncertain benefits for thrombolysis or surgery. Even transfer to a higher-level hospital for treatment likely would not have improved the prognosis. Considering the above medical indications and the family’s explicit financial and ethical concerns, the decision was ultimately made to discontinue treatment and discharge the patient.

This case primarily reports an episode of abdominal aortic embolism (most likely caused by atrial fibrillation),highlighting the critical importance of the therapeutic time window for this condition. Early diagnosis and intervention within this window may yield favorable outcomes. This case also serves as a stark reminder to clinicians of the significant hazards associated with atrial fibrillation complications. While cerebral embolism is more common in atrial fibrillation, both cerebral and systemic embolisms profoundly impact patients’ future quality of life, underscoring the critical need for prevention and management. In summary, for patients with atrial fibrillation, the following should be implemented: *early identification*: early detection through preliminary screening methods such as physician pulse palpation and cardiac auscultation. *Systematic diagnosis and treatment*: including management of comorbidities and risk factors, prevention of stroke and thromboembolism, symptom reduction through control of ventricular rate and rhythm, assessment and dynamic evaluation to maximize quality of life and improve prognosis.^[9]^

## Author contributions

**Conceptualization:** Tong Liu, Yuanyuan Che.

**Supervision:** Yuanyuan Che.

**Writing – original draft:** Tong Liu.

**Writing – review & editing:** Yuanyuan Che.
